# Resorbable plating system stabilizes tissue‐engineered intervertebral discs implanted ex vivo in canine cervical spines

**DOI:** 10.1002/jsp2.1031

**Published:** 2018-08-30

**Authors:** Jorge A. Mojica‐Santiago, Gernot M. Lang, Rodrigo Navarro‐Ramirez, Ibrahim Hussain, Roger Hӓrtl, Lawrence J. Bonassar

**Affiliations:** ^1^ Meinig School of Biomedical Engineering Cornell University Ithaca New York; ^2^ Weill Cornell Brain and Spine Center, Department of Neurological Surgery Weill Cornell Medicine, New York‐Presbyterian Hospital New York New York; ^3^ Department of Orthopaedic and Trauma Surgery Medical Center‐Albert Ludwig University of Freiburg, Faculty of Medicine Freiburg Germany; ^4^ Sibley School of Mechanical and Aerospace Engineering Cornell University Ithaca New York

**Keywords:** biomechanics, imaging, preclinical animal model, tissue engineering

## Abstract

Total disc replacement using tissue‐engineered intervertebral discs (TE‐IVDs) may offer a biological alternative to treat radiculopathy caused by disc degeneration. A composite TE‐IVD was previously developed and evaluated in rat tail and beagle cervical spine models in vivo. Although cell viability and tissue integration into host tissue were promising, significant implant displacement occurred at multiple spinal levels. The goal of the present study was to assess the effects of a resorbable plating system on the stiffness of motion segments and stability of tissue‐engineered implants subjected to axial compression. Canine motion segments from levels C2/C3 to C5/C6 were assessed as intact (CTRL), after discectomy (Dx), with an implanted TE‐IVD only (PLATE−), and with a TE‐IVD combined with an attached resorbable plate (PLATE+). Segments under PLATE+ conditions fully restored separation between endplates and showed significantly higher compressive stiffness than segments under PLATE− conditions. Plated segments partially restored more than 25% of the CTRL motion segment stiffness. Plate attachment also prevented implant extrusion from the disc space at 50% compressive strain, and this effect was more significant in segments from levels C3/C4 when compared to segments from level C5/C6. These results suggest that stabilization of motion segments via resorbable plating assists TE‐IVD retention in the disc space while allowing the opportunity for implants to fully integrate into the host tissue and achieve optimal restoration of spine biomechanics.

## 1 INTRODUCTION

Intervertebral disc (IVD) degeneration is known to alter the stability and biomechanics of cervical spine motion segments while decreasing the foraminal canal through which nerves stem from the spinal cord, in the most severe cases. Cervical radiculopathy that leads to debilitating or excruciating neck pain in patients (63.5‐107.5 per 100 000) is often associated to these changes.^1^ First‐line treatments include physical therapy and pharmacologic regimens, but surgical intervention is indicated in refractory cases or when the spinal cord is severely compromised. Although standard surgical treatments for cervical radiculopathy and myelopathy involve anterior cervical discectomy and fusion (ACDF) of the diseased motion segment, loss of spine flexibility and reduction of segment range of motion after surgical treatment is suspected to contribute to the onset of adjacent segment disease (ASD).^1^, ^2^ Total disc replacement or arthroplasty has also been explored as an alternative treatment to the golden standard to preserve segment stability and motion. However, the efficacy of cervical disc arthroplasty (CDA) in reducing the incidence of symptomatic ASD remains under debate.^3^, ^4^ While there may be other factors such as progression of the underlying disc degeneration that influence the occurrence of ASD, the rates of secondary surgical procedures in patients with ACDF is higher than those who received a CDA.^5^ Nevertheless, interbody implants and artificial disc replacements^6^, ^7^ are subject to wear and generation of debris that may lead to implant dislodgement, osteolysis, and mechanical failure. As such, the importance of restoring segmental motion and native IVD function with minimal risks of implant revisions cannot be overlooked.

Tissue‐engineered implants have been investigated in the last decade as biological alternatives to traditional treatments for radiculopathy. Composite tissue‐engineered intervertebral discs (TE‐IVDs) that mimic the form and function of the native disc have been developed by employing diverse fibrous materials to constitute the annulus fibrosus (AF) and isotropic gels to recreate the nucleus pulposus (NP).^8^, ^9^, ^10^, ^11^, ^12^, ^13^, ^14^, ^15^ We have shown previously a composite TE‐IVD that leveraged the cell‐driven contraction of collagen type I gels around alginate cores that demonstrated promising results in vitro and in vivo in the murine caudal spine and the canine cervical spine.^9^, ^15^ Our implants restored near native function and tissue integration in rat tails up to 6 months, and stably implanted TE‐IVDs maintained cell viability and integration into host tissue in the canine model for 16 weeks. However, retention of TE‐IVDs in the disc space remained to be achieved in 50% of the implanted canine segments. Displacement of TE‐IVDs during surgery led to destabilization of the motion segment and collapse of the endplates resulting in conditions similar to disc degeneration when implants were displaced through the ventral side.

The benefit of employing a soft engineered implant with a relatively compliant mechanical integrity lies in the ability of the TE‐IVD to mature in a dynamically stimulated environment while facilitating integration with surrounding host tissues. The collagen scaffold that constitutes the AF in TE‐IVDs undergoes remodeling due to the metabolic activity of embedded cells and the organization of its fibers into a structure that mimics the native disc is enabled by the initial concentration and contractibility of the gel scaffold.^16^ Notably, TE‐IVDs were shown to increase collagen and proteoglycans content by a factor of 10 over the course of 6 months in vivo, during which time the ECM integrated into neighboring vertebrae and provided physiological levels of mechanical function to the motion segment as assessed from static and dynamic aggregate moduli.^9^ Furthermore, we observed that all TE‐IVDs at the C3/C4 level remained stable in the canine spine in vivo, while all TE‐IVDs implanted at C5/C6 were displaced likely due to variations in size and angle of the VBs.^15^ Based on this previous work, we have identified two main challenges contributing to segment instability after placement of TE‐IVDs: (1) mechanical robustness within the motion segment is limited because the implant needs to be immature to promote integration; (2) vertebral anatomy of motion segments varies by level and is suspected to affect the stability of implantation, which results in implant migration out of the disc space. Axial dynamic distraction using an external fixator alone and in combination with cell therapy has been shown to promote disc repair in rabbit IVDs.^17^, ^18^, ^19^ To achieve IVD implant retention and prevent collapse of the disc space, external fixation of the vertebral bodies (VBs) has also been shown to provide stability in rodent caudal spines.^20^, ^21^ Although the disc space height was maintained in these animal models, the implants were not exposed to physiologic loading, which was integral for TE‐IVD maturation, integration to host tissue, and restoration of mechanical function. A bio‐resorbable fixation system made of 85:15 polylactic‐co‐glycolic acid (PLGA) plates and screws (Rapidsorb, Depuy Synthes Co. Johnson & Johnson, West Chester, PA), widely used for cranio‐maxillofacial trauma, provides an alternative for temporary and gradually dynamic stabilization of spine motion segments. The PLGA in this commercially available stabilization system has been well characterized for its biocompatibility and resorption kinetics, and is FDA approved for in vivo reconstructive procedures.

To address these shortcomings, we asked whether TE‐IVD implantation assisted by a resorbable plating system restores motion segment stiffness and prevent implant extrusion under axial compression, thereby improving overall stability of the treated segment. This PLGA system has been rated by the manufacturer to retain 85% of its strength for up to 8 weeks, while its bulk resorption is expected to occur within 12 months. A resorbable plate can enhance short term mechanics and keep the implant in place while the engineered tissues mature; however, the ability of resorbable plates to stabilize motion segments in combination with an engineered implant has not been shown. Our objectives with this study were to evaluate the restoration of the compressive mechanics of motion segments with a combined treatment approach of TE‐IVD implanted with a PLGA fixation system and identify the ability of these resorbable plates to prevent implant extrusion.

## 2 METHODS

### 2.1 Cell isolation and TE‐IVD fabrication

We adapted the cell preparation methods from previously established protocols.^8^, ^9^ Briefly, we harvested cervical IVDs from three skeletally mature canine spines (18‐36 months of age, Marshall BioResources, North Rose, NY), washed them in phosphate‐buffered saline (Dulbecco's PBS, MediaTech, Manassas, VA) with 1% antibiotic‐antimycotic solution (AbAm, 100 μg/mL penicillin, 100 μg/mL streptomycin, and 2.5 μg/mL amphotericin B, MediaTech), and diced the nucleus pulposus (NP) and annulus fibrosus (AF) tissue regions separately. After digesting NP and AF tissues in 0.3% wt./vol. collagenase type II (Worthington Biochemical Corp., Lakewood, NJ) at 37°C for 12 hours, we filtered the digested tissue solutions through a 100 μm nylon mesh (BD Biosciences, Bedford, MA). Subsequently, we cultured the NP and AF cells separately in Ham's F‐12 media (MediaTech) containing 10% fetal bovine serum (Gemini BioProducts, Sacramento, CA), 1% AbAm, and 25 μg/mL ascorbic acid (Sigma‐Aldrich, St. Louis, MO) to confluence.

Similarly, we based the TE‐IVD fabrication process on established techniques.^9^, ^15^ First, we mixed encapsulated canine NP cells (25 × 10^6^ cells/mL) in 3% wt./vol. alginate (FMC BioPolymer, Philadelphia, PA) two‐to‐one with a 0.02 g/mL calcium sulfate (Sigma‐Aldrich) solution. Then, we injected the mixture into customized 3D‐printed molds with cylindrical cavities made of acrylonitrile butadiene styrene plastic on a Ultimaker 2+ (Ultimaker North America, Cambridge, MA) to produce tissue‐engineered NPs. After 1 hour of immersion in 60 mM calcium chloride (Sigma‐Aldrich), we removed and placed the engineered NPs in the center of each well of a 12‐well plate. Next, we mixed an acidic 6 mg/mL collagen stock solution prepared from rat tail tendon fibers (Sprague Dawley, 7‐8 weeks old, Pel‐Freez Biologicals, Rogers, AR) with a basic solution (10× PBS, 1 N sodium hydroxide, and 1× PBS), in which we seeded canine AF cells (2 × 10^6^ cells/mL) to obtain a final concentration of 4 mg/mL. Finally, we created tissue‐engineered AF layers by surrounding the engineered NPs with 1.5 mL of the resulting collagen/AF solution and allowing gelation at 37°C for 30 minutes. Following gelation, we added 1 mL of previously described culture media to each well and cultured the TE‐IVDs for 4 weeks with media changes twice a week. TE‐IVD implants were made of the same cylindrical shape with an elliptical cross‐section for all motion segment levels.

In addition, we also prepared a group of acellular TE‐IVDs by adapting the protocol for high density collagen preparation as described previously.^22^ Briefly, we prepared and mixed collagen gel stock solutions at 20 mg/mL from collagen type I of the previously described source with the corresponding basic formulation to obtain a final concentration of 10 mg/mL. Then, we poured the resulting neutralized collagen solution into each well of 24‐well plates and allowed gelation at 37°C for 30 minutes before removing 8‐mm biopsy punches to simulate mature TE‐IVDs. Thereafter, we maintained these collagen plugs in PBS bath until used for displacement tracking.

### 2.2 Motion segment preparation

**Figure 1:**
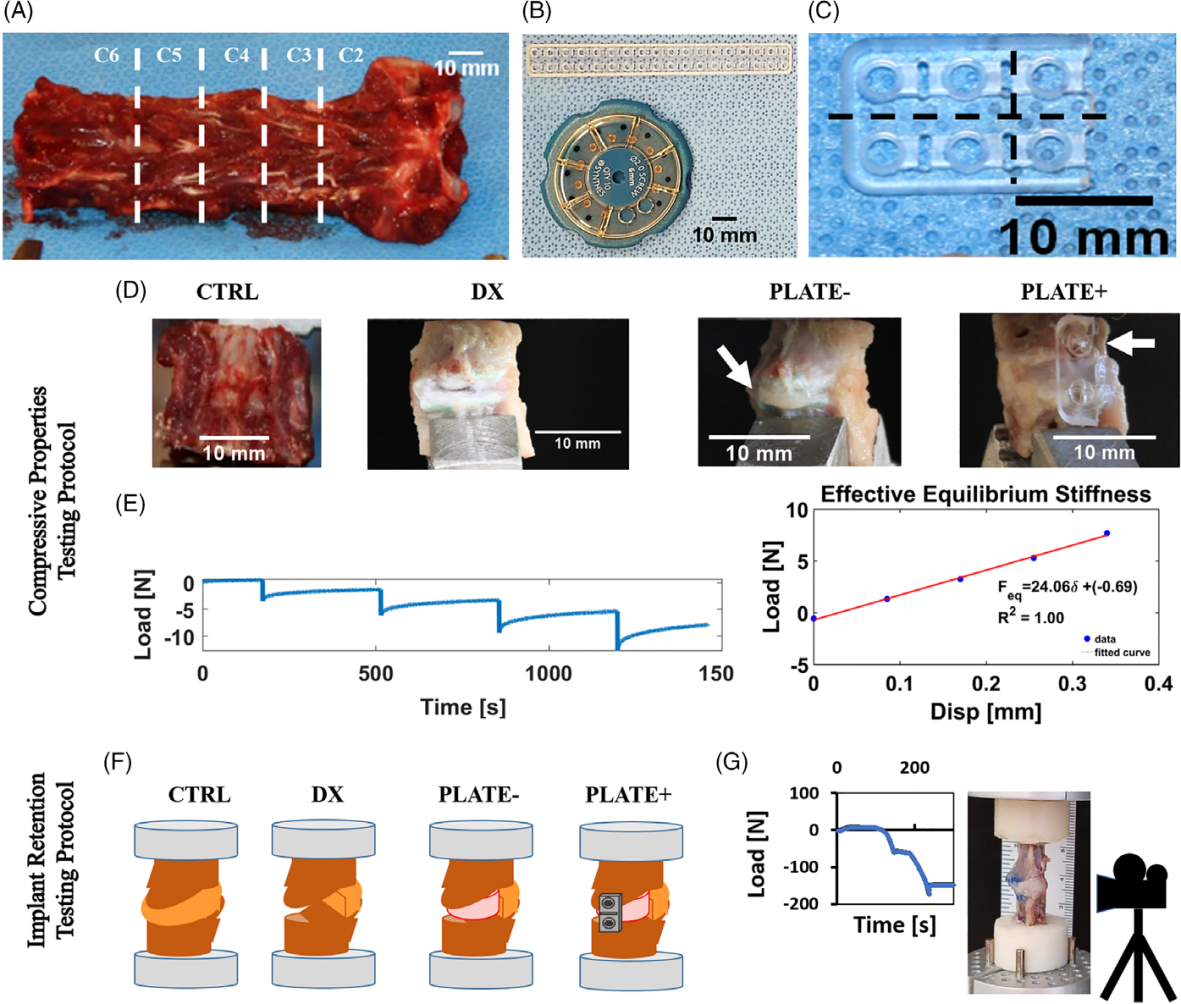
(A) Preparation of motion segment from levels C2/C3 to C4/C5 (dotted lines indicate vertebrae levels) for mechanical testing. (B) Close‐up image of PLGA plates and screws as supplied by manufacturer. (C) Close‐up detail of directions of cuts in PLGA plate (dotted lines). (D) Sample images of the motion segments under the examined conditions. (E) Testing protocol and setup used to assess the biomechanical response of a motion segment; curves show a sample of loading vs time for stress relaxation conditions and a sample of the resulting load vs displacement used to calculate segment stiffness. (F) Representation of the motion segments with VBs fixed in dental cement; PLATE− and PLATE+ depict prepared specimens for implant motion tracking. (G) Sample of a loading curve resulting from the constant strain rate protocol and setup used to track implant migration in a PLATE− specimen

To prepare specimens for testing, we obtained eight cervical spines of skeletally mature canines (18‐36 months of age, Marshall BioResources) and dissected motion segments from levels C2/C3 through C5/C6 by isolating the IVDs with the vertebral bodies on the adjacent cranial and caudal sides from all nerves, dorsal spinous processes, corresponding ligaments, and other soft tissues surrounding the IVD (Figure 1A). We kept the spines frozen after they were harvested from the donor animals and thawed them at room temperature before isolating the individual motion segments for further testing. We divided the specimens into two cohorts corresponding to the two distinct testing setups to test the biomechanical response of motion segments and to assess the implant retention in the disc space. For the first cohort of motion segments, we allocated four spines and isolated motion segments from levels C2/C3 to C4/C5 (*N* = 3 per spine) through bisection of each VB transversally such that each specimen comprised a native IVD with cranial and caudal endplates intact and half of its corresponding VBs cut (Figure 1D). For the second cohort, we isolated motion segments from the levels C3/C4 and C5/C6 of four spines (*N* = 2 per spine) by removing the adjacent C2/C3 and C4/C5 IVDs such that units of IVD with their corresponding cranial and caudal VBs remained intact. Afterwards, we embedded the VBs of these motion segments in dental molding cement (COE Tray Plastic, GC America, Alsip, IL) maintaining alignment of the long axis of the segment perpendicular to the top and bottom ends of the potting molds (Figure 1F).

We tested each specimen in each of the following conditions: (1) as intact (CTRL); (2) after discectomy (DX); and with an implanted TE‐IVD (3) without a resorbable plate (PLATE−) or (4) with the plate (PLATE+) (Figure 1D). Following initial testing of intact segment, we performed a standard discectomy making a box‐like incision through the ventral side and along the IVD/endplate interface followed by AF/NP extraction while preserving the posterior longitudinal ligament. After testing specimens under DX conditions, we inserted 4 mg/mL TE‐IVDs into empty disc space of the first cohort and acellular 10 mg/mL TE‐IVDs for the second cohort. We prepared fixation plates by cutting longitudinally along the centerline of an 85:15 PLGA plate of 2 × 18‐2.0 mm holes (Rapidsorb Rapid Resorbable Strut Plate, Depuy Synthes Co., West Chester, PA) and trimming transversally every two holes into fragments that appropriately matched the distance between endplates on the ventral side of each segment (Figure 1B,C). Since displacement of TE‐IVDs occurred ventrally in vivo, we aimed to apply the smallest possible plate that minimized the profile on the cervical spine motion segment. Following implantation of TE‐IVDs and testing on all specimens, we sanded the cranial and caudal endplates on the ventral side of each segment to fit the resorbable plate closely to the VB. We secured the plate at the ventral midline of each specimen with two 85:15 PLGA screws measuring 2 mm diameter by 6.0 mm long (Rapidsorb Rapid Resorbable Cortex Screw, Depuy Synthes Co., West Chester, PA), after drilling and tapping holes through the endplates, one in each of the VBs (Figure 1D). We chose not to mount wider plates or larger screws, because they would require a more invasive resection of the bony parts of the VBs upon implantation and could interfere with soft tissue structures surrounding the motion segments under in vivo conditions.

### 2.3 Biomechanical testing

We implemented two separate testing protocols: (1) multi‐step stress relaxation tests to measure the biomechanical response of motion segments under unconfined compression; and (2) continuous compression at constant strain rate to assess the migration of the implanted TE‐IVD. First, we took measurements of the VB dimensions, the outer IVD diameter, and disc height with calipers on the CTRL specimens. For the VB, we measured the distance between the contour of the endplate where the AF attaches and the edge of the VB that was cut after isolation from the cervical spine, as well as the major and minor axes of the cranial and caudal VBs. For all experimental conditions, we considered half the average disc space between endplates at the outer AF as the nominal height. Furthermore, we assumed rigid body motion for the VB and endplate, and that the change in IVD area between testing conditions was negligible. As such, all the axial deformations that occurred under each testing condition were assumed to be in the IVD. Subsequently, we reported the average measured height of the outer AF for DX, PLATE−, and PLATE+ groups as ratios of disc space height to intact segment under each condition.

For the first protocol, we clamped the caudal VB portion of the specimen to the load cell on a mechanical testing system (ELF 3200, EnduraTech, Eden Prairie, MN), while an impermeable plate applied 5% compressive strain steps up to 15% strain on the cranial VB portion (Figure 1E).^9^, ^23^ During each of the intact and experimental conditions described above, we kept the specimens surrounded by a gauze soaked with PBS (MediaTech) containing protease inhibitors (Roche Diagnostics, Indianapolis, IN). From the resulting load‐displacement data, we calculated an effective stiffness for the motion segment in equilibrium, and data from DX, PLATE−, and PLATE+ groups were normalized against their corresponding CTRL segments to calculate ratio of stiffness to intact segments under each condition.

For the second protocol, we clamped the potted VB of the specimen on its caudal end to the testing frame, while an impermeable plate compressed uniaxially at 0.5% strain/sec until segment collapse (Figure 1G). To track migration of acellular TE‐IVDs in segments, we recorded the uniaxial compression tests at 30 frames per second. We used a video camcorder (Sony CX440 Handycam, Sony Corp. of America, New York, NY) fixed on a tripod and controlled exposure settings and frame to focus on the disc space between endplates (Figure 3A).

### 2.4 Image analysis and digital image correlation

We matched the frames of the resulting videos to the compression test at constant strain rates and selected the frames corresponding to 5% strain until collapse (Videos S1 and S2, Supporting Information). Then, we used open source digital image correlation software (Ncorr v.1.2)^24^ to quantify two‐dimensional displacements at the region of interest (ROI) within the disc space corresponding to the TE‐IVD and the remaining AF tissues (Figure 3A). From the radial (horizontal) and axial (vertical) displacement maps (Figure 3B), we computed average magnitudes of the resultant displacement vectors in the ROI at discrete distances along the segment diameter between the ventral and dorsal sides of the disc space. To discretize the disc space, first we normalized the horizontal values of the ROI to this segment diameter and centered nominal radial locations around the mid‐axis of the disc space (*x* = 0). Then, we reported the mean displacements at each nominal radial location as the average of the values from the cranial endplate (*y* = 0) to the caudal endplate (*y* = disc space height) while excluding the empty background. We chose to compare the average magnitudes of PLATE− and PLATE+ experimental groups at 50% strain, since these were the maximum allowable strain of the intact motion segments corresponding to these groups (Figure 3C,D, Videos S3 and S4).

### 2.5 Statistical analysis

We reported all data as mean ± SD and evaluated data distribution in boxplots. For the biomechanical analysis, we conducted a repeated measures analysis of variance to compare the effect of segment level (C2/C3 to C4/C5) on the ratio of segment stiffness to intact segment in equilibrium over the experimental conditions DX, PLATE−, and PLATE+. We then used Tukey honest‐significance difference post‐hoc tests to identify significant differences at *P* < 0.05, with the Statistics and Machine Learning Toolbox of MATLAB R2017 (Mathworks, Natick, MA). For the image processing data, we used R (R‐Studio, Boston, MA) and the *lme4* function^25^ to perform a linear mixed effects analysis of the relationship between vector displacement magnitudes and treatment at discrete radial locations of un‐plated and plated segments. As fixed effects, we considered treatment (PLATE− vs PLATE+), nominal radial location (*x* = −1 on ventral side to *x* = 1 on dorsal side), and segment level (C3/C4 vs C5/C6) along with the two‐factor interaction terms for treatment. As random effects, we accounted for intercepts for spine (*N* = 4) and for the interaction between spine and level. We identified significant differences at *P* < 0.05 using Tukey adjustments for multiple comparisons.

## 3 RESULTS

### 3.1 Disc space height restoration

**Figure 2:**
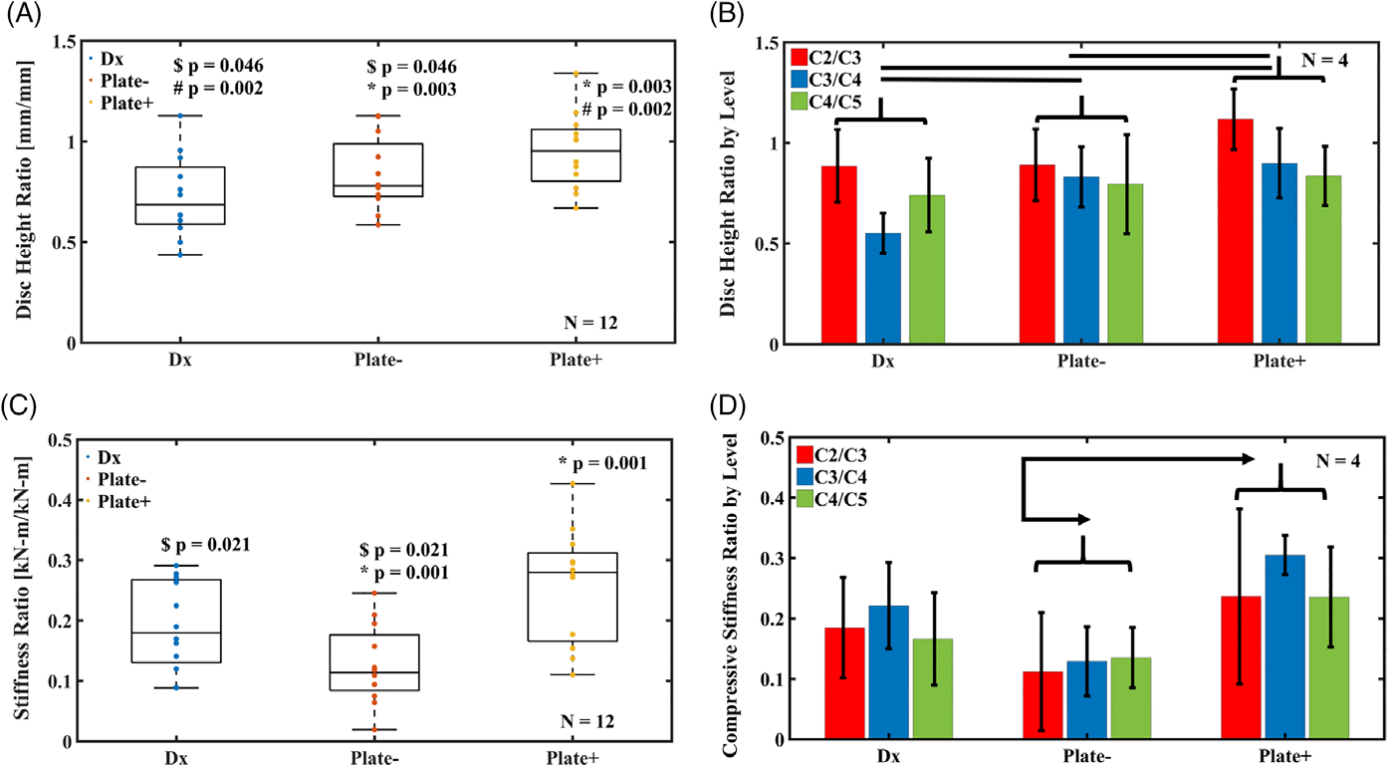
(A) Disc space height changes under the examined conditions for all motion segments normalized to their corresponding intact CTRL segments; $ *P* < 0.05 between DX and PLATE−, # *P* < 0.05 between DX and PLATE+, and * *P* < 0.05 between PLATE− and PLATE+. (B) Disc height ratio for motion segments at each level between C2 and C5 grouped by testing condition (lines correspond to the pairs of conditions with *P* < 0.05). (C) Compressive stiffness of all motion segments for each experimental condition normalized to their corresponding intact CTRL segments; $ *P* < 0.05 between DX and PLATE−, and * *P* < 0.05 between PLATE− and PLATE+. (D) Compressive stiffness ratio for motion segments at each disc level between C2 and C5 grouped by testing condition (arrows signal the pairs of conditions with *P* < 0.05). Boxplots show the data of all segment levels combined and their distribution through their median and quartiles, while bar graphs display data for each level as mean ± SD

Motion segments with implanted acellular TE‐IVDs and resorbable plates attached on the ventral side recovered initial height of the disc space before loading. Motion segments under PLATE+ condition reached significantly higher disc height than either DX (*P* = 0.002) and PLATE− (*P* = 0.003). While there were no marked differences in changes of disc space height between levels C2/C3, C3/C4, and C4/C5 (Figure 2B), the disc space height of all motion segments dropped by almost 30% when discectomized compared to the CTRL condition. TE‐IVD implantation alone increased the disc space height ratio to 0.84 ± 0.18, while plating in addition to the implant recovered up to 134% of original disc height (Figure 2A).

### 3.2 Motion segment stiffness preservation

In equilibrium, plating partially restored segment stiffness to more than 25% of the intact motion segment magnitudes of 60.9 ± 30.9 kN/m. Segments in PLATE+ group showed a significant two‐fold increase in stiffness (*P* = 0.001) when compared to the PLATE− group (Figure 2C). The stiffness of segments in DX group dropped by more than 80% of their CTRL stiffness and the stiffness ratio of PLATE− group segments to CTRL decreased even further at 0.13 ± 0.07. Segment stiffness ratios in PLATE+ and DX groups were statistically similar, despite the notable increase in disc height and more than 41% difference between their stiffness ratio to CTRL. Differences between stiffness ratio to CTRL were not significant across C2/C3, C3/C4, and C4/C5 levels (Figure 2D).

### 3.3 Improved implant retention

**Figure 3:**
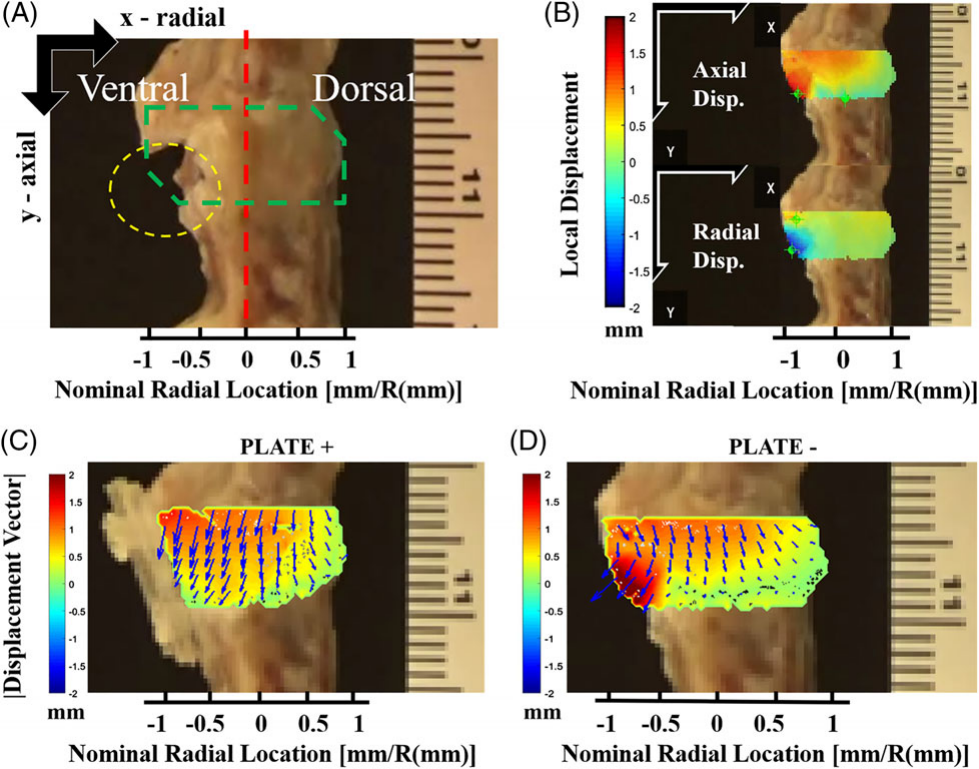
(A) Representative frame used to track implant migration in a motion segment and reference axes for the positive convention in the radial and axial directions; red dotted line shows central axis used to distinguish ventral and dorsal sides of the frame, green dotted line encloses the ROI within the disc space where displacement was computed, and yellow dotted ellipse encircles the sub‐region where TE‐IVD was located. (B) Samples of 2D displacement maps for magnitudes in the axial (top) and radial (bottom) orientations; color intensity values represent the local displacements in mm following the positive sign convention displayed by the axes in each of the corresponding orientations. (C) Representative plated segment with resultant vectors of displacement (blue arrows) and magnitudes (colormap). (D) Representative un‐plated segment with resultant vectors of displacement (blue arrows) and magnitudes (colormap). Combined vector fields and colormaps of displacements follow the established signed convention (positive downwards axially and to the right radially)

Attaching the plate prevented extrusion of the implant through the ventral side of all motion segments at 50% strain. The average magnitudes of vector displacements in the ROI were markedly affected by the nominal location along the radial direction (*P* < 2.2 × 10^−6^), by the treatment groups PLATE+ against PLATE− (*P* = 6.1 × 10^−7^), and by the combined interactions of treatment with segment level (*P* = 1.3 × 10^−9^). Notably, the average displacements in the disc space remained below 0.6 mm in the dorsal side, while the average displacements occurring in the ventral side exceeded 1.1 mm. The specific region located between 30% distance from the center in the ventral side and 100% distance from the center in the dorsal side (dorsal edge) delimited where average displacements were significantly lower across all treatments and levels. The maximum displacements of the acellular TE‐IVDs recorded in the PLATE− group were observed consistently at the caudal endplate near the extrusion site (Figure 3D, Video S3), while the maximum displacements in the PLATE+ group were distributed along the cranial endplate (Figure 3C, Video S4).

**Figure 4:**
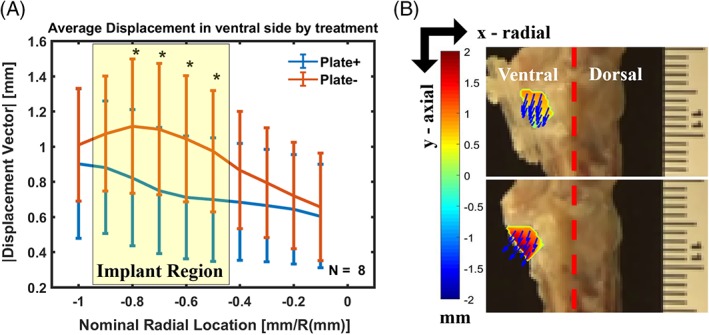
(A) Processed displacement vector magnitudes measured in the ventral side of the selected ROI in all C3/C4 and C5/C6 motion segments; yellow box corresponds to the region where the TE‐IVD was located; * *P* < 0.05 represent significant differences between PLATE− and PLATE+ at the corresponding radial location; data are shown as mean ± SD. (B) Representative frames of PLATE+ (top) and PLATE− (bottom) with resulting displacement vector fields and magnitude colormaps specifically corresponding to the implant migration (outlined in yellow)

Plating reduced implant migration between 8% and 32% (Figure 4(A)) at discrete locations of the disc space. Segments from PLATE+ group had significantly lower average displacement magnitudes than those from PLATE− group at nominal radial locations between 80% and 50% distance from the center on the ventral side, corresponding to the region where the implant was located (Figures 3C,D and 4B). We observed that implants were partially expelled from the disc space by 5% to 10% strain and 15% to 25% strain in PLATE− segments, at C5/C6 and C3/C4, respectively. This trend was consistent with the statistical inference that plating was more effective in reducing implant migration at C3/C4 segments (*P* < 0.0001), than in C5/C6 segments (*P* = 0.41) (Table S1). Nevertheless, different levels had no significant fixed effects on average displacements at the ROI. There were also no significant random effects observed between spines and combining spines with levels.

## 4 DISCUSSION

In the present study, we tested the hypothesis that resorbable plating improved stiffness of canine cervical spine motion segments in vitro and prevented the extrusion of implanted TE‐IVDs from the disc space. We demonstrated that the combination of TE‐IVDs implanted with PLGA plates reconditioned motion segment stiffness in compression by restoring the disc space height after discectomy and stabilizing the segment, while retaining the implant in place. Previous efforts of total disc replacement with a combined approach of tissue‐engineered implants and external fixation focused on preventing segment collapse and implant displacement.^20^ However, there has been no previous work demonstrating the ability of an implantable bio‐resorbable fixation system to restore motion segment stability while preserving disc space height and retention of an engineered implant.

The PLGA temporary stabilization system adequately addresses our findings in the in vivo canine model of cervical disc replacement,^15^ where some TE‐IVDs displaced ventrally. While implanted TE‐IVDs alone were able to retain up to 70% of healthy control disc height in motion segments in vivo and more than 80% of intact CTRL disc height in vitro (Figure 2A,B), only with the attachment of the plate we observed complete recovery of disc space height. The relative rigidity of the PLGA plate compared to the hydrogel‐based TE‐IVDs and the fixation of the PLGA screws through the endplates contributed to the increase in disc height after discectomy. Since attachment of the PLGA screws was performed in an angular fashion from the sanded surface on the ventral side of the caudal and cranial endplates through the VBs, the rigid straight plate was fit closely to the implanted TE‐IVD, thus effectively restoring distraction of the VB in the PLATE+ group before loading. In preliminary studies, TE‐IVDs demonstrated apparent equilibrium moduli in compression that ranged in the 0.5 to 5 kPa, which remains orders of magnitude lower than the 0.03 to 5.96 MPa apparent equilibrium modulus observed in the intact cervical spine segments gathered ex vivo. Meanwhile, the PLGA copolymer that constitutes the plates and screws has an initial elastic modulus of 3.1 GPa and an ultimate tensile strength of 66 MPa.^26^ The combined approach in PLATE+ segments achieved sufficient mechanical robustness to partially restore intact CTRL segment stiffness (Figure 2C,D) when compared to PLATE− segments; therefore, this partial mechanical support allows for continuous load sharing and dynamic mechanical stimulation to the TE‐IVD, while transferring loads through the endplates. Furthermore, preliminary tests of cervical spine segments without resecting the posterior elements and soft tissues revealed that between 60% and 80% of compressive loads applied onto intact motion segments are shared by these additional tissues. The relative similarity of the intact stiffness ratio between DX and PLATE+ groups likely results from the contact between endplates after DX which confounds the rigidity of an empty disc space with that of a treated segment.

The resorbable plate is expected to provide temporary structural biomechanical support while promoting implant integration in the first 4 to 16 weeks, since the PLGA plate degrades between 4 and 12 months.^27^ Faster degradation kinetics in vitro than in human maxillofacial bones in vivo have been shown previously with PLGA implants; however, the stiffness of intact explants upon isolation from the surrounding fibrous tissue and bone could not be tested.^26^ Due to the slower degradation observed clinically in this case, the copolymer implant was expected to retain its bending strength for longer periods than the 75 days tested in vitro. It should be noted that the contraindications of the Rapidsorb fixation system warn against its use in load‐bearing applications, unless in conjunction with traditional rigid fixation. In human patients, a combined ACDF approach of a polyether ether ketone spacer with resorbable materials such as poly(L‐lactide‐co‐D,L‐lactide) has been shown to provide similar fusion progress and stability than traditional titanium fixation;^28^ however, our intent with this study was to provide temporary stabilization to the implanted TE‐IVD instead of promoting rigid fixation of the motion segment. In this context, degradation of the PLGA system is expected to promote a gradual change in load distribution between the TE‐IVD and the PLGA plate, unlike the case of existing interbody spinal fusion techniques and external fixation devices previously evaluated in human cadaveric spines.^29^, ^30^, ^31^, ^32^ Mackiewicz et al confirmed in a finite element study that introducing highly stiff stabilizing plates into cervical spine motion segments increases stress in the endplate of adjacent segments and that plates that allow greater range of motion show up to 30% reduction of adjacent plates resulting stress.^33^ Matge et al.^34^ discussed clinical and radiological observations that suggest dynamic cervical implants as a promising alternative to total disc replacement, anterior cervical discectomy and spinal fusion, and they indicate that preserving motion segment biomechanics reduces stress on facet joints and development of adjacent segment disease. The advantage of our combined approach over existing interbody cage designs and dynamic cervical prosthetics remains in that our TE‐IVD has been shown to remodel over time and mature to restore mechanical function of spine segments to native conditions, when stably implanted and fully engrafted.^9^

The use of video recorded frames during the uniaxial continuous compression protocol and digital image correlation for data processing enabled the quantitative analysis of implant migration within the disc space. Acellular TE‐IVDs were retained within the disc space of all segments under PLATE+ conditions, because the PLGA plates served as a physical barrier that prevented complete extrusion of the implants. The distribution of maximum displacements along the cranial endplate in PLATE+ segments (Figure 3C) suggests a shift of the implant to accommodate to the endplate shape on the cranial side. As expected, the location of maximum displacements of the PLATE− segments occurred at the implantation site where the space with least resistance remained open (Figure 3D), since the inclined shape of the endplate in the ventral side combined with the lack of anchoring for the implant resulted in a wedge‐like extrusion. These observations were consistent with the mechanism of extrusion observed in the displaced implants of our in vivo canine study.^15^ The significant reduction of implant migration in PLATE+ at locations in the ventral side further supports the ability of resorbable plating to retain the TE‐IVD inside the disc space (Figure 4). Furthermore, the relatively similar displacement profiles observed in all segments outside of the 50% to 80% range of radial distance from central axis suggests that spine flexibility and overall range of motion around the location of the implant remains unaffected. The quantitative analysis of implant migration also revealed level‐dependent differences in the efficacy of our combined treatment approach. Displacements of TE‐IVDs were more effectively reduced at C3/C4‐level PLATE+ segments than in those of levels C5/C6 (Table S1), likely due to the anatomic differences at the endplates of both segments. These differences were also reflected in vivo where 66.67% of the stably implanted TE‐IVDs were located in C3/C4‐level segments, while 100% of the TE‐IVDs implanted in the C5/C6‐level segments were displaced.^15^ In human cervical spines, marked anatomic differences exist between superior and inferior endplates at upper level and lower level segments.^35^, ^36^ These findings provide further insights into the careful considerations that need to be taken when deciding location of implants in cervical motion segments. Whereas the reported measurements of displacement are limited to the resultant sum of local deformations caused by the applied load from those caused by rigid body motion, these parameters provide a quantitative estimate of segment motion under uniaxial compression. Future studies could benefit of differentiating texture to enhance segmentation of the implant and the surrounding tissues within the image and multiple projections to capture a three‐dimensional range of motion.

Several limitations in this work warrant important discussion when interpreting our findings within the context of an ex vivo model of an in vivo scenario. First, this study only assesses the compressive stiffness uniaxially, which does not recapitulate accurately what occurs in the physiological environment in vivo. However, our motivation for uniaxial mechanical testing was based on the intraoperative observations where the implants migrated out of the disc space solely from the deformations applied in axial compression upon removal of the distractor pins.^15^ Furthermore, with the assistance of the surrounding muscles and ligaments, individual cervical spine segments in quadrupeds are mainly loaded under axial compression to balance the bending moments from the weight of the head and neck.^37^ Challenges remain in characterizing the biomechanical response of motion segments with our proposed treatment under cyclic loading or fatigue, both of which are also relevant in spine biomechanics and help inspect modes of failure in the system. Future work should investigate dynamic testing of motion segments and their biomechanics in the full six degrees of freedom characteristic of the spine. Second, while favorable outcomes in terms of durability and minimal negative inflammatory responses have been shown in cranio‐maxillo‐facial approaches with several animal models using PLGA plates and screws,^38^, ^39^ their load‐bearing capacity remains limited.^40^ Recent work by Maenz et al.^41^ with PLGA‐reinforced calcium phosphate cement in ovine VBs suggests a potential use in load‐bearing structures given the versatility and manufacturability of PLGA. Third, this study does not include a direct assessment of the degradation kinetics of the resorbable system and its effects on the load distribution across the VBs over time. While in vitro assessment of the degradation of PLGA system could provide estimates on strength retention, the use of this resorbable systems in future in vivo work is preferable to appropriately assess the efficacy of our proposed combined approach. Fourth, our surgical approach and modifications to the plate require further tuning to minimize the need for sanding the endplate and altering the VB profile. Since the ACDF approach is characterized by minimal invasiveness compared to dorsal or lateral fixation, we recommend exploring the attachment of customized PLGA elements that can adapt to the curvature of the ventral side of the motion segment. Finally, anatomical differences between human and canine cervical motion segments could also have significant impact on the performance of the resorbable plate examined in this work; furthermore, canine IVDs are exposed to similar or even higher loading compared to humans.^37^, ^42^, ^43^, ^44^, ^45^, ^46^, ^47^ However, the canine spine shows analogous degenerative processes to those of humans and are regularly diagnosed and treated for disc degeneration with equivalent surgical approaches.^37^, ^46^, ^47^

This study provides valuable insights of canine motion segment biomechanics and validates a combined approach of total disc replacement with TE‐IVD and an implantable resorbable fixation system. Our findings in the current study present a baseline for further ex vivo and in vivo animal studies to better discern the long‐term biomechanical and integrative properties of cervical TE‐IVDs stabilized by resorbable plating. In addition, the method herein described to quantify displacements at specific locations along the radius of endplate offers a tool for estimation of loads occurring at the disc in diverse surgical scenarios. This work demonstrates that the combination of an implanted TE‐IVD with a resorbable plate improves implant retention by preventing ventral displacement under uniaxial compression, partially restores the compressive stiffness of intact segments by providing a shared distribution of loads, and helps avert the collapse of endplates in the treated disc space.

## Supporting information


**Video S1** Supplementary material

**Video S2** Supplementary material

**Video S3** Supplementary material

**Video S4** Supplementary material

**Table S1** Summary of vector displacement magnitude averaged over the entire range of nominal radial locations from the ventral side to the dorsal side (x = −1:1 mm/R[mm]) and the resultant *P* values from the multiple comparison tests at 0.95 confidence level. Date are grouped by treatment (Plate− vs Plate+, and by level C3/C4 vs C5/C6); linear mixed model analysis revealed significant difference between treatments at level C3/C4 only (* *P* < 0.05)
