# Supplementary figures and images for: Resorbable plating system stabilizes tissue‐engineered intervertebral discs implanted ex vivo in canine cervical spines
Source: JOR Spine. 2018 Aug 30;1(3):e1031. doi: 10.1002/jsp2.1031 (PMC6686817; doi:10.1002/jsp2.1031)

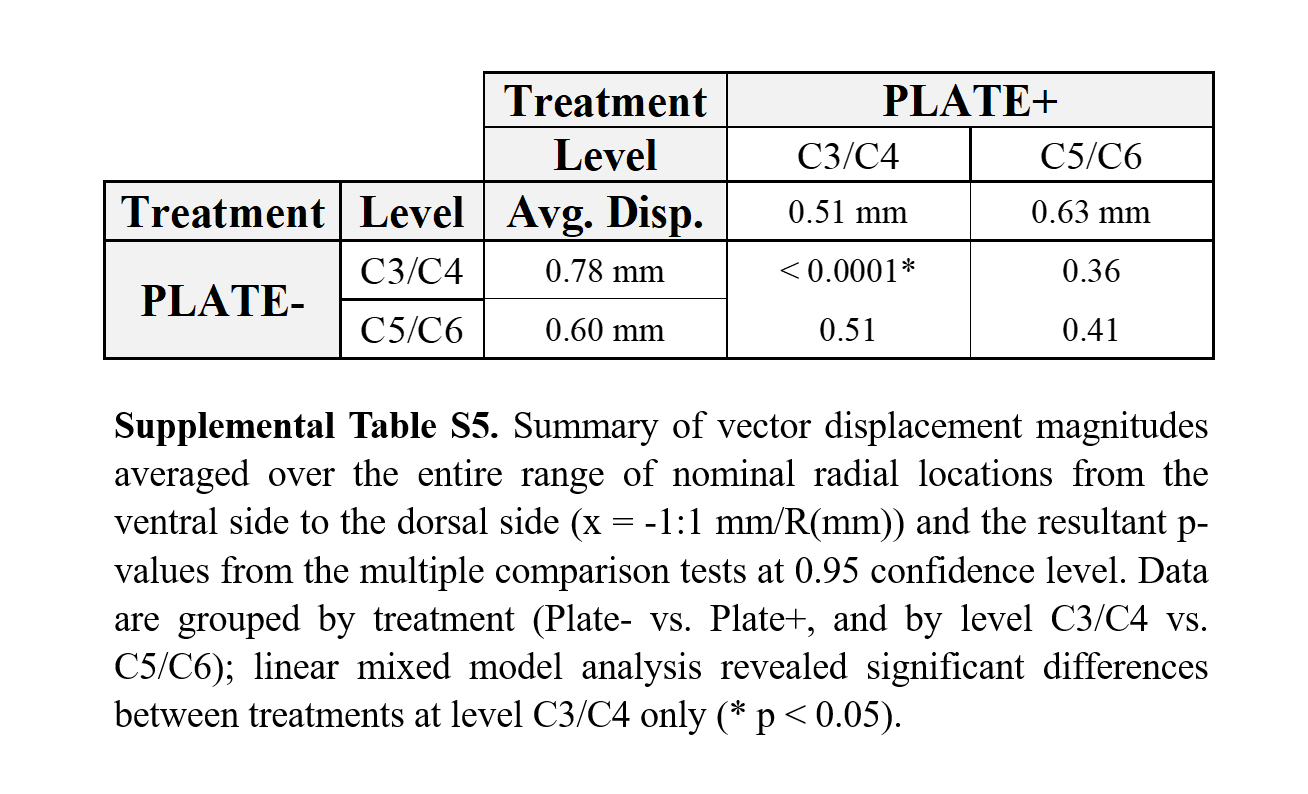

Supplement: Supplementary file 5 — Table S1 Summary of vector displacement magnitude averaged over the entire range of nominal radial locations from the ventral side to the dorsal side (x = −1:1 mm/R[mm]) and the resultant P values from the multiple comparison tests at 0.95 confidence level. Date are grouped by treatment (Plate− vs Plate+, and by level C3/C4 vs C5/C6); linear mixed model analysis revealed significant difference between treatments at level C3/C4 only (* P < 0.05) [file JSP2-1-e1031-s003.tif]
